# Supplementary material for: Comparing the Efficacy of Two Generations of EGFR-TKIs: An Integrated Drug–Disease Mechanistic Model Approach in EGFR-Mutated Lung Adenocarcinoma
Source: Biomedicines. 2024 Mar 21;12(3):704. doi: 10.3390/biomedicines12030704 (PMC10968545; doi:10.3390/biomedicines12030704)
Supplement: Supplementary file 1 [file biomedicines-12-00704-s001.zip › biomedicines-2890105-supplementary.pdf]

Supplementary Materials: Comparing the Efficacy of Two Generations of EGFR-TKIs: An Integrated Drug–Disease Mechanistic Model Approach in EGFR-Mutated Lung Adenocarcinoma

Hippolyte Darré [1], Perrine Masson [1], Arnaud Nativel [1], Laura Villain [1], Diane Lefaudeux [1], Claire Couty [1], Bastien Martin [1], Evgueni Jacob [1], Michaël Duruisseaux [2,3,4], Jean-Louis Palgen [1], Claudio Monteiro [1], Adèle L'Hostis [1*]

S1. Development of the ISELA-V2 model

The ISELA-V1 model is composed of three pillars: (i) a disease model, (ii) a treatment model ; (iii) a virtual population. Each of these pillars was expanded to broaden the context of use of the model: this new model version is named ISELA-V2.

- The disease model was enhanced to bring credibility to the model: as detailed in L'Hostis et al. [1], neoangiogenesis was identified through sensitivity analysis as a phenomenon that impacts tumor growth. For this reason, we decided to detail it more. The paper also identified the immune system as phenomena that impacts the model, this is an additional improvement that could be added in the future
- Cell cycle and cell death was also added to represent in greater detail the link between EGFR downstream activation and tumor cell growth and survival.
- An osimertinib PBPK model was added, as detailed in the paper and in the supplementary data.
- The virtual population was expanded to consider patients with metastasis

S1.1. Disease model enhancement

Mechanistic models of the following phenomena were plugged in ISELA-V1 disease model to enhance it:

- A cell cycle and cell death model was implemented to represent the evolution of the layer of proliferative cells in LUAD tumor tissues
- A neo-angiogenesis model was developed to follow the dynamic evolution of tumor-induced vascular network, allowing the study of individual endothelial cell subpopulations and their contribution to the overall tumor carrying capacity.

S1.1.1. Cell cycle and cell death

The scope of the model is to represent the evolution of the layer of proliferative cells in a LUAD tumor tissue. Cancer proliferation through the cell cycle is governed by cyclin-dependent kinases (Cdks), with their activities regulated by a complex network sensitive to internal and external factors [2].

- G1 phase progression is triggered by D- and E-type cyclins partnering with CDKs, amplifying cyclin expression through positive feedback, while regulatory proteins like p16, p21, and p27 halt CDK activity if DNA damage occurs [3].
- S phase requires CDK2 activation for DNA replication, influenced by Cyclin A and regulated by the ATR/CHK1 pathway in response to replication issues [4].
- G2 phase advances to M phase via the CDK1/CyclinB complex, with CHK1 and WEE1 ensuring no progression in case of DNA issues [5].
- M phase involves CDK1 activation by CDC25 phosphatase, with the APC/C complex finalizing cell division post-SAC, signaling cyclin degradation [6].

This phenomena is represented in Figure S1.

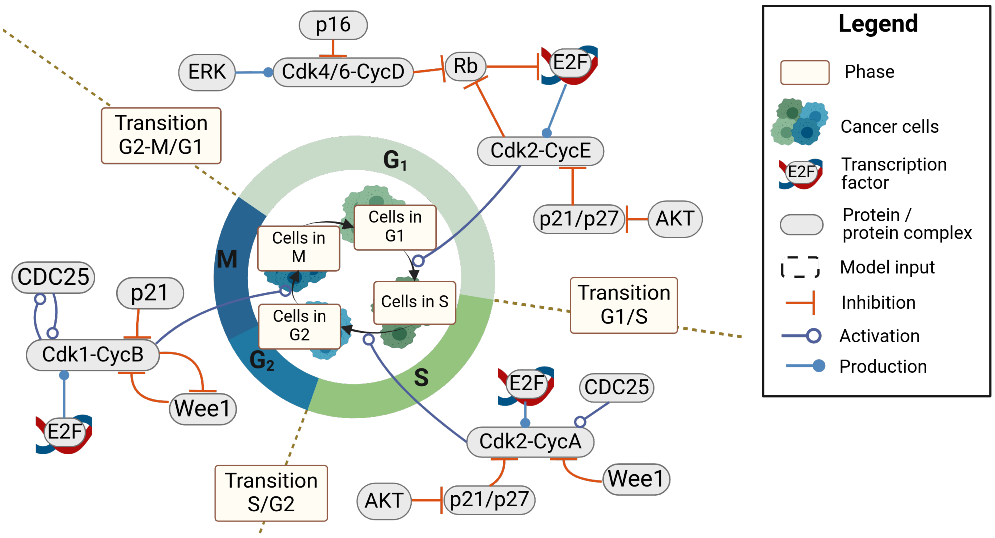

Figure S1. Schema of the cell cycle and cell death in ISELA-V2. Created with Biorender.com, 2024.

S1.1.2. Neoangiogenesis

The neoangiogenesis submodel aims at modeling the dynamic evolution of tumor-induced vascular networks. Mechanistic models allow the study of individual endothelial cell subpopulations and their contribution to the overall tumor carrying capacity and metastases dynamics. It considers three main phenomena:

- Local dynamics of tumor angiogenesis factors: indeed, while under normal conditions, a balance between pro-angiogenic and anti-angiogenic functions is maintained by endothelial cell receptors, hypoxic tumors disrupt this balance by releasing tumor angiogenic factors (TAFs), including VEGF, leading to increased neoangiogenesis [7]. VEGF, the most studied pro-angiogenic factor, is mainly produced by tumor and endothelial cells, promoting cell proliferation, migration, and increased vascular permeability, with a self-amplifying loop observed in non-small cell lung cancer (NSCLC), particularly lung adenocarcinoma (LUAD), where high VEGF levels correlate with poor prognosis [8][9]. VEGF expression is primarily regulated by hypoxia through hypoxia-inducible transcription factors (HIFs), with the ERK and AKT pathways modulating HIF-1α in NSCLC, indicating a critical role for hypoxia in neoangiogenesis induction [10].
- Endothelial cells (EC) dynamics: the EC pool can be either functional (mature) or non-functional (immature), with their development driven by TAFs, which can independently increase, decrease, or stabilize the EC pool based on their effective step and interactions.
- Impact on the tumor: neoangiogenesis governs the amount of tumor cells that may receive enough oxygen to survive. In this submodel, we consider this aspect, by assuming that a constant fraction of these receive enough oxygen to proliferate. The carrying capacity or maximal load is defined as the maximum tumor cell population size that can be sustained by the environment given the resources and in particular oxygen. SInce proliferative cells need more resources, two carrying capacities could be defined, one for survival and one for proliferation.

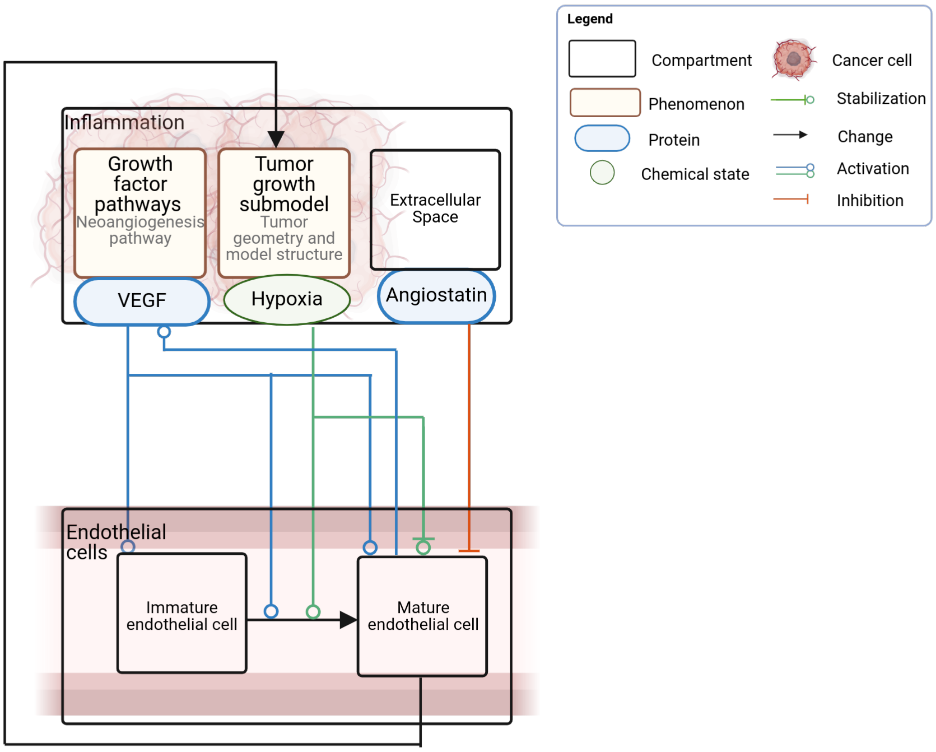

Figure S2. Schema of the neoangiogenesis in ISELA-V2. One tumor is able to synthesize both VEGF, through the EGFR downstream pathway, and other TAFs, described by hypoxia. These TAFs have several impacts on the endothelial cells dynamics (synthesis, maturation, stabilization and deletion). Also, the mature endothelial cells pool modifies the carrying capacities of the tumor. Metastases are impacted by the main tumor TAF but also have their own neoangiogenesis processes. Created with Biorender.com, 2024.

S1.2. Treatment model additions

As detailed in the Material & Methods section, osimertinib and gefitinib PBPK treatment models were then added, as well as the emergent mechanisms of resistance observed following osimertinib administration (the mechanisms of resistance to gefitinib were already implemented in ISELA-V1). Osimertinib and gefitinib submodel are respectively detailed in Figure S3 and Figure S4.

S1.2.1. Osimertinib

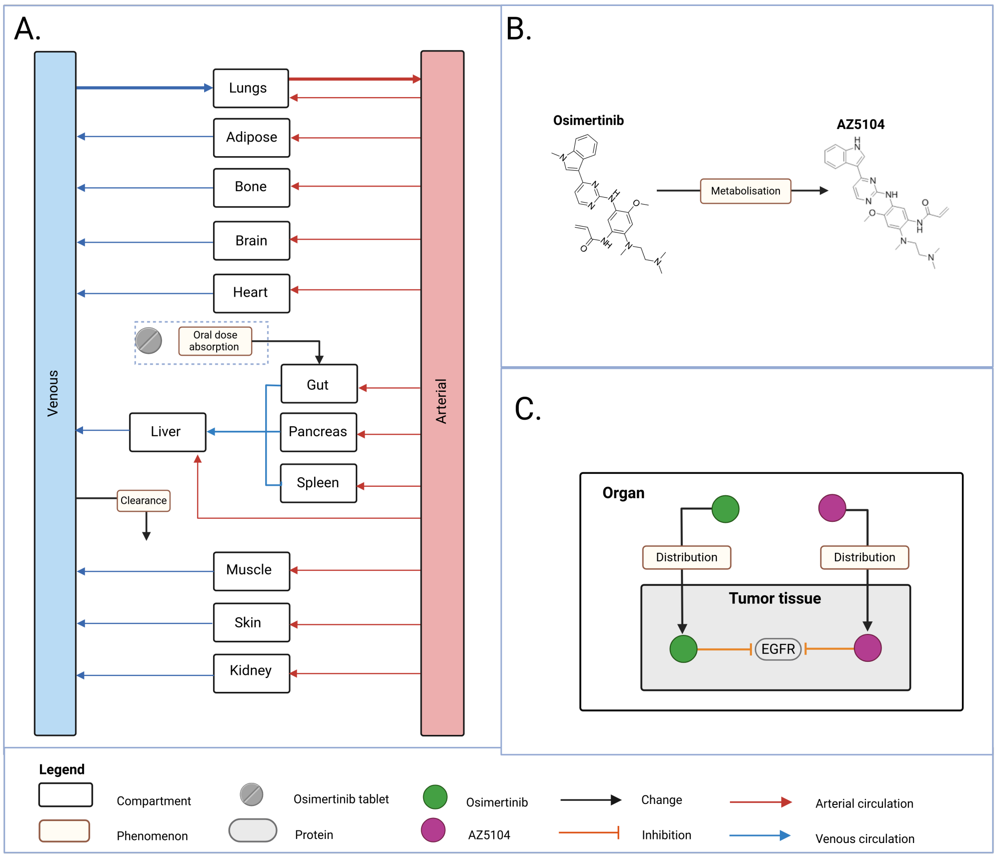

Figure S3. Representation of the osimertinib treatment submodel.(**A**) Pharmacokinetic model of osimertinib. (**B**) Metabolization of osmertinib in AZ5104. (**C**) Mechanism of action of osimertinib and AZ5104. Created with Biorender.com, 2024.

S1.2.2. Gefitinib

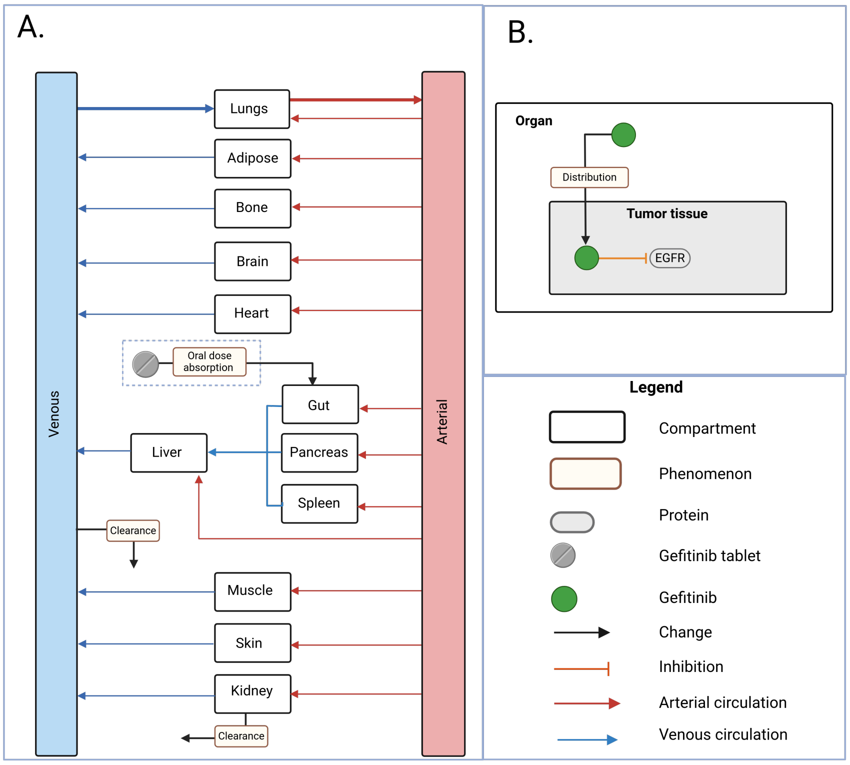

Figure S4. Representation of the gefitinib treatment submodel.(**A**) Pharmacokinetic model of gefitinib. (**B**) Mechanism of action of gefitinib. Created with Biorender.com, 2024.

S1.3. Virtual population expansion

Finally, the virtual population of the ISELA-V1 model was expanded to overcome a limiting hypothesis: that the metastatic state of the patients remains the same. By duplicating the disease model enhanced from ISELA-V1 for each potential metastase, ISELA-V2 can describe the emergence and growth of LUAD metastases in parallel to the primary lung tumor, and the impact of EGFR-TKIs on secondary tumors. The driving hypothesis is that the growth of each modeled metastasis (MT) follows the same model as the primary tumor (PT), with specific adaptations related to local distinctive characteristics and initial size.

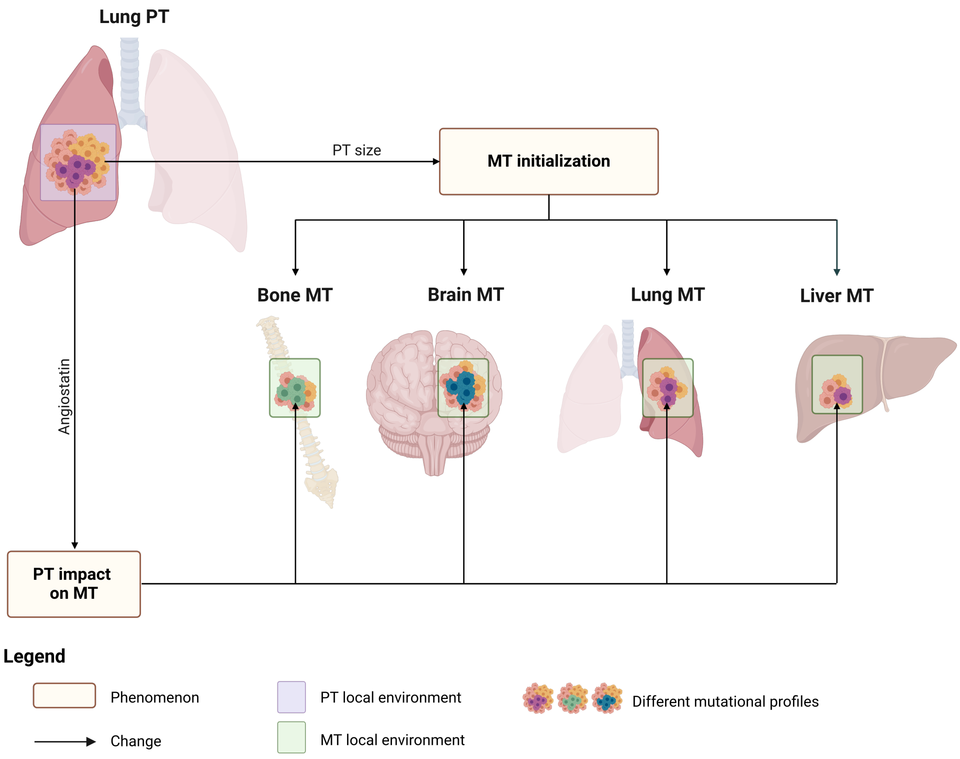

Figure S5. Schema of the metastases in ISELA-V2. Created with Biorender.com, 2024.

S1.4. Graphical illustration of the ISELA-V2 model

ISELA-V2 model is illustrated hereafter:

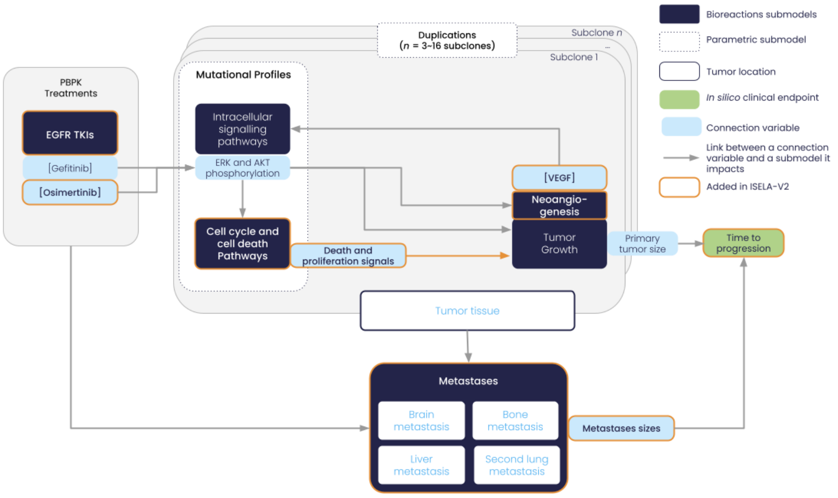

Figure S6. Structure of the ISELA-V2 model: the different submodels are labeled and their connecting variables are represented in light blue. The two main model outputs are also represented (i) the biological one, corresponding to the radius of the primary & metastases tumors; (ii) the clinical one, corresponding to the time at which the disease progressed, as defined according to the RECIST (Response Evaluation Criteria In Solid Tumors) guidelines (version 1.1) [11].

S2. Structure of the PBPK model

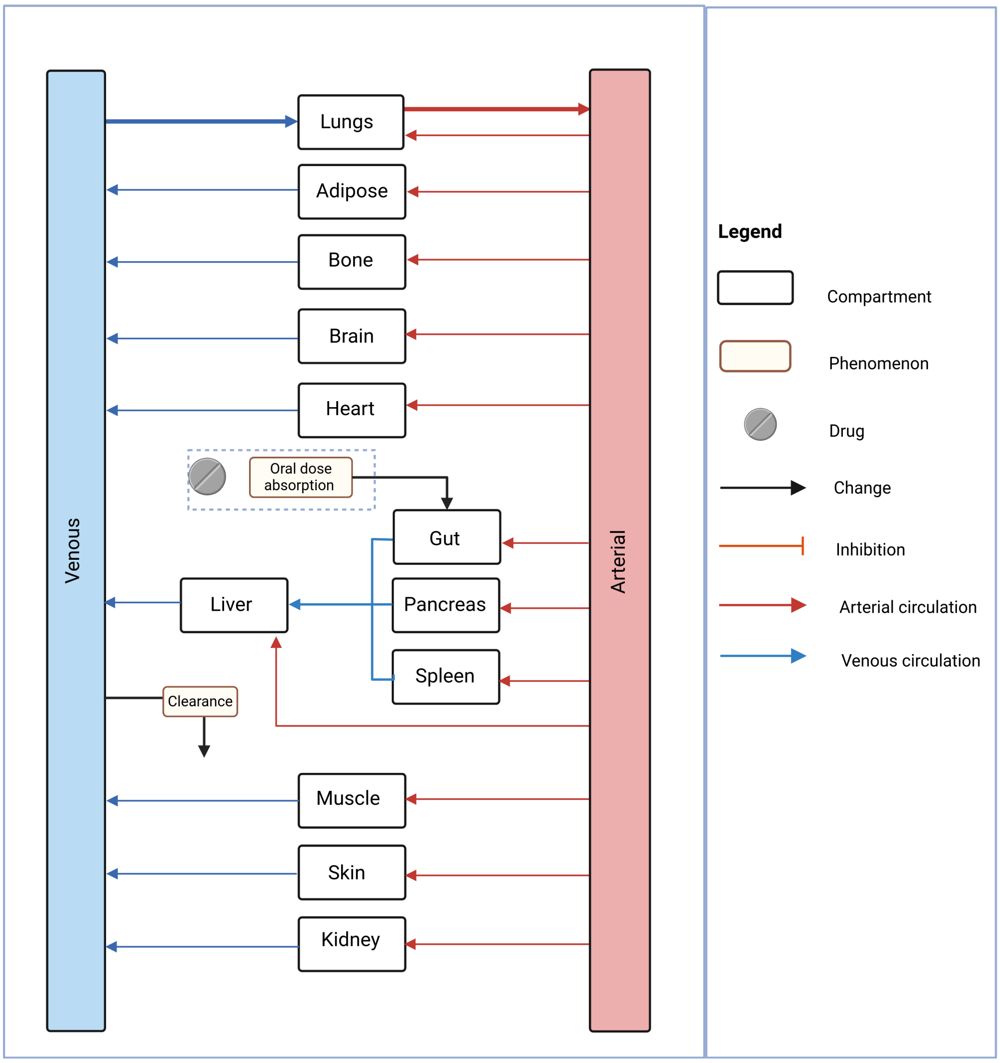

Figure S7. Representation of the PBPK structure used to build osimertinib and gefitinib PBPK models. Created with Biorender.com, 2024.

PBPK models are pharmacokinetic models which predict the concentration over time of a drug in multiple tissues and fluids by explicitly taking into consideration tissue physiology and anatomy as well as drug physico-chemical properties and biochemistry. This knowledge is used to predict the drug's interactions with the organism in terms of its absorption, distribution, metabolism and excretion (ADME). Once those phenomena are modeled using a mechanistic representation of physiological processes, the concentration-time profile of the drug can be established in multiple compartments, including the one where the drug elicits its pharmacodynamic action. The PBPK model is composed of a number of organs where the drug can be distributed. Each organ is represented as a compartment with its anatomical and physiological properties. To each compartment of the model is associated a blood flow rate, a volume and a tissue partition coefficient. The compartments in the model are linked by the arterial and venous compartments. The Figure S7 represents the different organs modeled and their impact on the drug. To include the differences observed in physiology across age and gender, the weight of the organs and their associated blood supply are age and sex dependent with the values being taken from the ICRP 23 [12].

S2.1. Absorption

Mechanisms regulating absorption after oral administration are multiple, including diverse mechanisms as drug disintegration and dissolution, degradation, gastric emptying, intestinal transit, intestinal permeation, intestinal and hepatic metabolism, and can be quite complex, as they are governed by the properties of both the compound (pKa, lipophilicity) and the gastrointestinal tract (gastrointestinal pH, gastric metabolism, etc.) [13]. However, in our approach, as we already have the pharmacokinetic data in humans and it is not the objective to predict the drug concentration in a different context than the recommended dose, we implemented a simple absorption model inspired by classical PK models. We added a symbolic compartment called "Administration" where the drug is placed at time of administration. This amount of drug is equal to the drug dose multiplied by a factor called $k_{FractionAbsorbed}$ to account for the known bioavailability of the drug. Then the drug will diffuse to the gut compartment modeled with a mass action kinetic law.

$$\mathbf{Drug_{Administration}} \overset{k_{Absorption} * Drug_{Administration}}{\longrightarrow} \mathbf{Drug_{Gut}} \tag{1}$$

S2.2. Distribution

Drug distribution is the reversible partitioning of a drug from the systemic blood circulation into the different tissues of the body. It is driven by blood flow rates or the ability of drugs to cross membrane barriers. It leads to defined proportions in the different tissues at steady-state. Drug distribution occurs for each and every administration route. There seems to be a consensus on distribution models emerging from the literature. The subsequent equations take into account the hypothesis that organs are considered well-stirred compartments. A well-stirred compartment is a compartment in which the drug concentration is uniform and any incoming drug is instantaneously distributed. Under the well-stirred assumption, distribution of a drug into a compartment can be modeled as rate-limited by one of two processes: perfusion or permeability. In every organ, the hypothesis that the rate was limited by perfusion was made. Perfusion-rate limited kinetics occur for many small-molecules and lipophilic drugs and mean that the rate of distribution is only limited by blood flow. It is assumed that drugs can diffuse easily and rapidly into the interstitial and intracellular spaces and that unbound drug concentrations are equal on each side of the cell membrane [14].

$$\mathbf{Drug_{Arterial}} \overset{Q_{Compartment} * \frac{Drug_{Arterial}}{vol(Arterial)}}{\longrightarrow} \mathbf{Drug_{Compartment}} \tag{2}$$

$$\mathbf{Drug_{Compartment}} \overset{Q_{Compartment} * \frac{Drug_{Compartment}}{vol(Compartment) * CorrectedKp} * R}{\longrightarrow} \mathbf{Drug_{Venous}} \tag{3}$$

With

$$CorrectedK_p = f_{VS}^X + K_p^S * K_p^X * (1 - f_{VS}^X) \tag{4}$$

And

- R: blood to plasma concentration ratio
- f_{VS}^X: fractional volume of vascular space in organ X
- K_p^X: tissue-to-plasma ratio in organ X, calculated following the distribution theory proposed by Rodgers And Rowland [15]
- K_p^S: Kp scalar which is a fitted factor common to all Kp

The exception to those equations being the liver as it receives blood from the arterial circulation as well as the gut, pancreas and spleen:

$$\begin{aligned}\frac{dDrug_{Liver}}{dt} = {} & QLiver_{In} * \frac{Drug_{Arterial}}{vol(Arterial)} + QGut * \frac{Drug_{Gut} * R}{vol(Gut) * CorrectedKp_{Gut}} \\ & + QSpleen * \frac{Drug_{Spleen} * R}{vol(Spleen) * CorrectedKp_{Spleen}} \\ & + QPancreas * \frac{Drug_{Pancreas} * R}{vol(Pancreas) * CorrectedKp_{Pancreas}} \\ & - QLiver_{Out} * \frac{Drug_{Liver} * R}{vol(Liver) * CorrectedKp_{Liver}}\end{aligned} \tag{5}$$

S2.3. Elimination/Metabolism

The general goal of metabolism is to render molecules more polar and hydrophilic to make them more easily excreted in urine [16]. In our PBPK models, we grouped the metabolism and elimination of the drug under a venous plasmatic clearance of the drug.

$$\mathbf{Drug_{Venous}} \overset{k_{Clearance} * Drug_{Venous}}{\rightarrow} \emptyset \tag{6}$$

The only exception is osimertinib for which one of its metabolites (AZ5104) is active and present in a non-negligible concentration with respect to osimertinib and for which the metabolization was modeled. The following reaction represents the metabolization of osimertinib to AZ5104:

$$\mathbf{OsimertinibVenous} \overset{k_{Clearance} * OsimertinibVenous * fraction_{Metabolisation}}{\rightarrow} \mathbf{AZ5104_{Venous}} \tag{7}$$

Note that to conserve the quantity of drug, the plasmatic clearance of osimertinib has been multiplied by (1-fractionMetabolisation). The fraction of metabolization to AZ5104 has been informed by the literature and is equal to 0.25 in humans [17] and 0.7 [18] in mice.

The parameters that have been calibrated in order to reproduce the pharmacokinetic data are: $k_{clearance}$, $k_{absorption}$, $k_{FractionAbsorbed}$ and K_p^S for gefitinib, osimertinib and AZ5104.

Abbreviations

The following abbreviations are used in this manuscript:

ADME	Absorption Distribution Metabolism Elimination
AKT	Protein Kinase B
DNA	Deoxyribonucleic acid
EGFR	Epidermal Growth Factor Receptor
ERK	Extracellular signal-regulated kinase
ISELA	In Silico EGFR Lung Adenocarcinoma
LUAD	Lung Adenocarcinoma
MT	Metastasis
PK	Pharmacokinetics
PBPK	Physiologically based pharmacokinetics
PT	Primary tumor
RECIST	Response Evaluation Criteria In Solid Tumors

References

1. Adèle L'Hostis; Jean-Louis Palgen; Angélique Perrillat-Mercerot; Emmanuel Peyronnet; Evgueni Jacob; James Bosley; Michaël Duruisseaux; Raphaël Toueg; Lucile Lefèvre; Riad Kahoul; Nicoletta Ceres; Claudio Monteiro, Knowledge-based mechanistic modeling accurately predicts disease progression with gefitinib in EGFR-mutant lung adenocarcinoma. *npj Syst Biol Appl* **2023**, *9*, 37
2. Rajat Singhania; R. Michael Sramkoski; James W. Jacobberger; John J. Tyson, A Hybrid Model of Mammalian Cell Cycle Regulation. *PLos Comput Biol.* **2011**, *7(2)*

3. Su Su Thae Hnit; Chanlu Xie; Mu Yao; Jeff Holst; Alan Bensoussan; Paul De Souza; Zhong Li ; Qihan Dong, $p27^{Kip1}$ signaling: Transcriptional and post-translational regulation. *The International Journal of Biochemistry and Cell Biology.* **2015**, *68*, 9-14
4. Stein, G.S.; Medina, R.; van Wijnen A.J.; Stein, J.L.; Lian, J.B.; Owen, T.A., Cell cycle. https://onlinelibrary.wiley.com/doi/10.1002/9780470015902.a0001354.pub2 *eLS. John Wiley and Sons Ltd.*
5. Maria Castedo; Jean-Luc Perfettini; Thomas Roumier; Karine Andreau; Rene Medema; Guido Kroemer, Cell death by mitotic catastrophe: a molecular definition. *Oncogene.* **2004**, *23(16)*, 2825-37
6. Andrea Ghelli Luserna di Rorà; Claudio Cerchione; Giovanni Martinelli; Giorgia Simonetti , A WEE1 family business: regulation of mitosis, cancer progression, and therapeutic target. *J Hematol Oncol.* **2020**, *13(1)*, 126
7. Roy M Bremnes; Carlos Camps; Rafael Sirera, Angiogenesis in non-small cell lung cancer: the prognostic impact of neoangiogenesis and the cytokines VEGF and bFGF in tumours and blood. *Lung Cancer.* **2006**, *51(2)*, 143-58
8. Ming-Szu Hung; I-Chuan Chen; Paul-Yann Lin; Jr-Hau Lung; Ya-Chin Li; Yu-Ching Lin; Cheng-Ta Yang; Ying-Huang Tsai, Epidermal growth factor receptor mutation enhances expression of vascular endothelial growth factor in lung cancer. *Oncol Lett.* **2016**, *12(6)*, 4598-4604
9. S. Vazquez; U. Anido; M. Lazaro; L. Santome; J. Afonso; O. Fernandez; A. Martinez de Alegria L. A. Aparicio, Angiogenesis and Lung Cancer. *Oncogenesis, Inflammatory and Parasitic Tropical Diseases of the Lung* **2013**, *1*
10. Diane R Bielenberg; Bruce R Zetter, The Contribution of Angiogenesis to the Process of Metastasis. *Cancer J.* **2015**, *21(4)*, 267-73
11. E.A. Eisenhauer et al. , New response evaluation criteria in solid tumours: Revised RECIST guideline (version 1.1). *European Journal of Cancer* **2009**
12. ICRP, Report of the Task Group on Reference Man. ICRP Publication 23. Pergamon Press, Oxford. **1975**
13. Huang W, Lee SL, Yu LX, Mechanistic approaches to predicting oral drug absorption. *AAPS* **2009**, *11(2)*, 217-24.
14. Mayumi K, Ohnishi S, Hasegawa H., Successful Prediction of Human Pharmacokinetics by Improving Calculation Processes of Physiologically Based Pharmacokinetic Approach. *J Pharm Sci* **2019**, *108(8)*, 2718-2727.
15. Rodgers T, Rowland M., Physiologically based pharmacokinetic modelling 2: predicting the tissue distribution of acids, very weak bases, neutrals and zwitterions. *J Pharm Sci* **2006**, *95(6)*, 1238-57.
16. Nikhil TaxakPrasad V Bharatam, Drug Metabolism: A Fascinating Link Between Chemistry and Biology. *Resonance Journal of Science Education* **2014**, *19(3)*, 259-282.
17. Brown K, Comisar C, Witjes H, Maringwa J, de Greef R, Vishwanathan K, Cantarini M, Cox E., Population pharmacokinetics and exposure-response of osimertinib in patients with non-small cell lung cancer. *Br J Clin Pharmacol.* **2017**, *83(6)*, 1216-1226.
18. James W.T. Yates; Susan Ashton; Darren Cross; Martine J. Mellor; Steve J. Powell; Peter Ballard, Irreversible Inhibition of EGFR: Modeling the Combined Pharmacokinetic–Pharmacodynamic Relationship of Osimertinib and Its Active Metabolite AZ5104 . *Mol Cancer Ther* **2016**, *15(10)*, 2378-2387.

Disclaimer/Publisher's Note: The statements, opinions and data contained in all publications are solely those of the individual author(s) and contributor(s) and not of MDPI and/or the editor(s). MDPI and/or the editor(s) disclaim responsibility for any injury to people or property resulting from any ideas, methods, instructions or products referred to in the content.
